# Female Health Workers at the Doorstep: A Pilot of Community-Based Maternal, Newborn, and Child Health Service Delivery in Northern Nigeria

**DOI:** 10.9745/GHSP-D-14-00117

**Published:** 2015-03-02

**Authors:** Charles A Uzondu, Henry V Doctor, Sally E Findley, Godwin Y Afenyadu, Alastair Ager

**Affiliations:** a Partnership for Reviving Routine Immunization in Northern Nigeria–Maternal Newborn and Child Health Initiative, Kano, Nigeria; b Columbia University, Mailman School of Public Health, New York, NY, USA

## Abstract

**Introduction:**

Nigeria has one of the highest maternal mortality ratios in the world. Poor health outcomes are linked to weak health infrastructure, barriers to service access, and consequent low rates of service utilization. In the northern state of Jigawa, a pilot study was conducted to explore the feasibility of deploying resident female Community Health Extension Workers (CHEWs) to rural areas to provide essential maternal, newborn, and child health services.

**Methods:**

Between February and August 2011, a quasi-experimental design compared service utilization in the pilot community of Kadawawa, which deployed female resident CHEWs to provide health post services, 24/7 emergency access, and home visits, with the control community of Kafin Baka. In addition, we analyzed data from the preceding year in Kadawawa, and also compared service utilization data in Kadawawa from 2008–2010 (before introduction of the pilot) with data from 2011–2013 (during and after the pilot) to gauge sustainability of the model.

**Results:**

Following deployment of female CHEWs to Kadawawa in 2011, there was more than a 500% increase in rates of health post visits compared with 2010, from about 1.5 monthly visits per 100 population to about 8 monthly visits per 100. Health post visit rates were between 1.4 and 5.5 times higher in the intervention community than in the control community. Monthly antenatal care coverage in Kadawawa during the pilot period ranged from 11.9% to 21.3%, up from 0.9% to 5.8% in the preceding year. Coverage in Kafin Baka ranged from 0% to 3%. Facility-based deliveries by a skilled birth attendant more than doubled in Kadawawa compared with the preceding year (105 vs. 43 deliveries total, respectively). There was evidence of sustainability of these changes over the 2 subsequent years.

**Conclusion:**

Community-based service delivery through a resident female community health worker can increase health service utilization in rural, hard-to-reach areas.

## Abstract (precis)

Deployment of resident female Community Health Extension Workers (CHEWs) to a remote rural community led to major and sustained increases in service utilization, including antenatal care and facility-based deliveries. Key components to success: (1) providing an additional rural residence allowance to help recruit and retain CHEWs; (2) posting the female CHEWs in pairs to avoid isolation and provide mutual support; (3) ensuring supplies and transportation means for home visits; and (4) allowing CHEWs to perform deliveries.

## INTRODUCTION

About 342,900 women worldwide died from causes related to pregnancy and childbirth in 2008.^1^ Sub-Saharan Africa bears a disproportionate burden of these deaths, with 3 of 5 of these deaths occurring in the region. Nigeria alone—with one of the highest maternal mortality ratios (MMRs) in the world—had an estimated 50,000 maternal deaths that year. Further, in contrast to many countries where the MMR has declined over recent decades, Nigeria is one of the few countries in the world where maternal mortality has shown no substantial reduction (516 per 100,000 live births in 1980 compared with 576 per 100,000 live births in 2013).^2^ Thus, the country has for some time been signaled as one that will fail to meet the Millennium Development Goal (MDG) of reducing maternal mortality by 75% by 2015.^3^ The situation is of particular concern in northern Nigeria, where the MMR is estimated to be significantly higher than the national average. Recent estimates for the northern region exceed 1,000 per 100,000 live births compared with MMR estimates for the southern region, which are below 300 per 100,000 live births.^4^^,^^5^

Maternal mortality in northern Nigeria is significantly higher than the national average.

In 2007, the Partnership for Reviving Routine Immunization in Northern Nigeria and the Maternal, Newborn and Child Health program (PRRINN-MNCH) was established, with support from the Department for International Development of the United Kingdom (DFID) and the Government of Norway, to address these issues in 4 states of northern Nigeria (Jigawa, Katsina, Yobe, and Zamfara).

In Jigawa state, the focus of the current study, a household survey had identified low rates of service utilization of basic primary health care services.^6^ For example, only 21.5% of women had reported having their last baby in a health facility, and 21.2% of children between 6 and 23 months of age had received the recommended 3 doses of polio vaccine. Challenges of access and transport were reported as particularly extreme. Nearly 3 of 4 (71.5%) women reported that transportation and related access issues had prevented them from accessing health care services (compared with 49.9%, on average, in all 4 northern states).

Transportation and related access issues impede health care service utilization in northern Nigeria.

Renewed interest in the potential role and contribution of community health workers has gained momentum in recent years.^7^^,^^8^ Many such cadres are community volunteers tasked with mobilizing community members to access the formal health system.^9^^–^^14^ However, other cadres include trained employees deployed to bring services closer to the “doorsteps” of mothers and children, either through home visits or by being readily available at a local health facility or in emergencies.^15^^–^^17^

The Community Health Extension Worker (CHEW) is a long-established cadre of health workers in Nigeria designed for this “doorstep” role. Schools of health technology in Nigeria offer either a 2- or 3-year CHEW training program, the latter featuring more training in the actual delivery of infants. However, CHEWs in Jigawa (and other states in the north) are some way from fulfilling the promise to extend services to their communities. Outreach to homes is not within the usual scope of their practice, and distribution of CHEWs has been largely within urban areas. Rural areas that are served usually have not had resident CHEWs, severely restricting the functional hours that residents can access the health center (and virtually eliminating emergency access). Additionally, with the significant gender gap in educational attainment in the region, the majority of the CHEWs are male. This furthers restricts functional access to services in the community given cultural barriers to women receiving care from men.

The lack of reach of CHEWs into most communities was recognized by Jigawa's state health officials as a major factor in the low service utilization rates of primary health care services, including critical maternal and newborn care. In the context of the health system strengthening goals of the PRRINN-MNCH program, these officials sought examples of strategies to invigorate such a cadre for more effective community engagement. Ghana's Community-Based Health Planning Service (CHPS) strategy, originally developed in Navrongo to bring services to especially remote communities,^21^ was recognized as a potentially relevant approach. Following a visit to Navrongo, Jigawa state health officials chose to pilot an adaptation of Ghana's CHPS strategy.^18^

The Navrongo CHPS model emphasizes the value of health workers living and working in the community where they are potentially able to provide truly community-based services. Nigerian CHEWs were considered broadly comparable to the community health officers and nurses of the Navrongo CHPS program, who had 18 months of training plus a 6-week orientation to community engagement and work based in the community.^19^ However, an important adaptation of the Navrongo, and existing Nigerian, model was to explore the recruitment of only female CHEWs for such community-based work.

In this article, we report on a pilot study that examined the feasibility and outcomes associated with implementing this model of female, community-based CHEWs providing essential MNCH services within one Local Government Area (LGA) of Jigawa state. The lessons learned were expected to inform decisions about potential expansion of this model within the LGA, to other LGAs in Jigawa, to other states—and, indeed, to other nations—facing similar constraints.

## METHODS

### Site Selection

Jigawa state is divided into 5 emirates—Dutse, Hadejia, Kazaure, Ringim and Gumel—each headed by an emir. For the pilot, we selected Jahun LGA, located 45 km from the state capital of Dutse and within the Dutse emirate. The Jahun Gunduma Council is the major governing structure for health provision in Jahun as well as Miga LGA, with responsibility for 30 primary health centers (PHCs) and Jahun General Hospital.

Jahun's population density of 152 persons/km^2^ is typical for the state, and was thus considered an appropriate context for the pilot with potential for scale-up to other LGAs. The close location of the state School of Health Technology—the institution that trains CHEWs—also provided logistical benefits for initial phases of the work focused on curriculum review and training.

According to 2006 census figures, Jahun LGA had a total population of 229,094 people.^20^ The specific target for this pilot were women of childbearing age, and particularly pregnant women. There were an estimated 57,000 women of childbearing age in Jahun LGA, with approximately 20% pregnant at any given time.

Within Jahun LGA, Kadawawa was selected to pilot the community-based service delivery program. Located approximately 36 km from the LGA headquarters, Kadawawa is a remote, rural community with a population of just under 16,000 people spread out across several hamlets. The terrain of the community is rough and barely accessible during the rainy season. Commercial vehicles and motorbikes get to Kadawawa once a week on market days. When health workers have to travel to and from the community, this is feasible only on market days, which fall on Fridays. Jahun General Hospital, approximately 1.5 hours away by vehicle, is the nearest basic or comprehensive obstetric care center to Kadawawa. Kadawawa is estimated to have a population of just under 3,000 women of childbearing age, with a little less than 800 pregnant women at any one time and some 600 women having children under 1 year of age. Before deployment of resident health workers, Kadawawa clinic was in a generally poor state of repair, serving only as a base for male CHEWs to provide immunization and related services on weekly visits to the community.

The community of Kafin Baka was selected as the control community because, although smaller in population, it otherwise had similar baseline characteristics to Kadawawa (Table 1). It is a rural village positioned within difficult terrain. It had a dilapidated and ill-equipped, although semi-functional, health center managed by non-resident male CHEWs who provided services on an irregular basis. As in Kadawawa, there were no female CHEWs at the health facility, and there were no outreach services by female health workers of any grade. The control site provided no ANC, deliveries, or postnatal care services, although the male health workers provided immunizations. The distance to Jahun General Hospital—the nearest basic/comprehensive obstetric care center—was similar to that for residents of Kadawawa, with similar transportation costs for the journey (around 5,000 Nigerian Nairas [NGN], or about US$25).

Transportation costs from the study communities to the nearest hospital were around US$25.

**Table 1.** Selected Demographic Characteristics for Kadawawa (Intervention) and Kafin Baka (Control) Communities in Nigeria at Baseline

	**No. (%)**	
	**Kadawawa**	**Kafin Baka**
Population	15,954	6,748
Population under 1 year old	638 (4%)	270 (4%)
Women of reproductive age	2,712 (17%)	1,485 (22%)
Pregnancies at any given time	789 (5%)	337 (5%)

During the study period, both the intervention and control sites received general facility upgrades through Jigawa's implementation of a minimum service package. This focused on equipment and supplies but did not include staffing changes related to CHEWs.

### Pre-Intervention Studies

Pre-intervention qualitative studies were conducted to inform study design and pilot implementation, as well as to mobilize the community prior to implementation, a key feature of the Navrongo CHPS model. These studies included mapping of undeserved areas and focus group discussions (FGDs), key informant interviews, and community dialogues (*durbars*) to identify perceptions of factors contributing to the high rates of maternal and newborn mortality (including low service utilization) within the communities of Kadawawa and Kafin Baka.^21^ FGDs were held with women of childbearing age, older women who had stopped childbearing, and young unmarried girls. Discussions were also held with older men, young married men, and young unmarried men, using similar FGD guides. Key informant interviews were held with community leaders (men, women, youth, and religious leaders). Community dialogues were convened to ensure potential inclusion of community members who had not participated in other forms of consultation. Other pre-intervention studies involved in-depth interviews with CHEWs and health managers regarding the current practices and deployment of CHEWS, as well as a review of the CHEW training curriculum at the School of Health Sciences.

**Figure f04:**
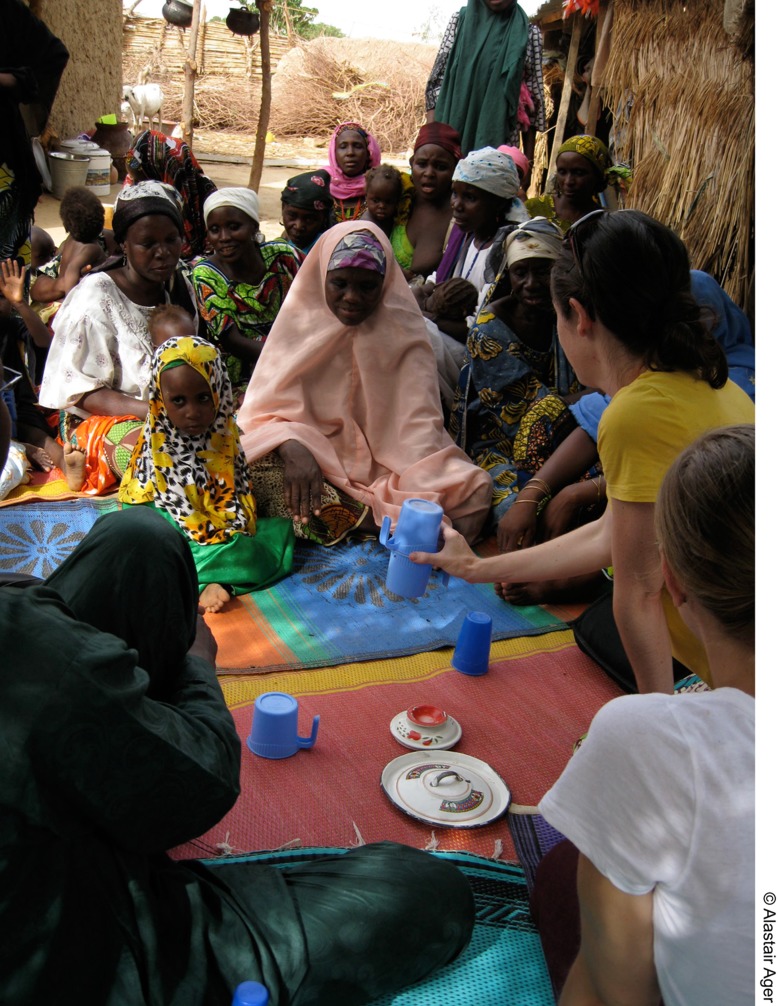
Consultations with community members helped shape the design of the pilot community-based service delivery program.

The pre-intervention qualitative research identified 3 major types of barriers to accessing existing services: household-level barriers, including cultural norms discouraging women's prompt access to care; facility-level barriers, largely lack of resources; and attitudinal issues, which further discouraged access to the referral facility in Jahun (Table 2).

**Table 2.** Summary of Pre-Intervention Qualitative Study Findings in Jigawa State, Nigeria

**Theme**	**Findings**
Barriers hindering pregnant women from accessing health care	• Women have to seek approval from an authority figure to access health care.
	• Weak women were perceived to deliver at the hospital and strong women at home.
	• Community members lacked knowledge about pregnancy danger signs.
Barriers at the community health facility level	• Poorly equipped health facilities: “We have to give birth on cement floors because there is only one bed …”
	• Limited access to drugs: “We need medicines, we need our hospitals to be repaired …”
	• Limited access to health personnel, especially female personnel: “The male CHEWs are afraid of touching us …”; “[Female CHEWs] should be available in the facility at all times, not coming from time to time …”
	• No emergency transport to Jahun General Hospital: “The roads are bad and the hospital a long distance away … transportation is too high [costly] and if the only available car has gone to the market, that is all …”
Barriers at the referral facility level	• Perceived negative outcomes to delivering at Jahun General Hospital: “If CHEWs referred us to Jahun General Hospital, we will not really be happy to hear that …”
	• Feeling insulted and harassed by staff at Jahun General Hospital: “They ignore us and call us villagers …”

In-depth interviews with CHEWs, health managers, and government leaders also identified a range of supply-side challenges, including a shortage of midwives, isolation, and problems in the operation of the “Midwives Service Scheme” (the national scheme deploying midwives to underserved communities). They also noted the circumstances that had resulted in CHEWs evolving from what were intended to be a community-based cadre to a clinic-based cadre. CHEWs were originally trained to spend 60% of their time within the communities doing home visits and 40% at designated rural health clinics. But their postings became increasingly politicized, and a majority of them were found living in urban areas and working in urban city clinics, avoiding rural areas. Men who married the few female CHEWs that had been trained in the state often did not allow their wives to live and work in rural areas.

The interviews with CHEWs and review of their training curricula indicated further barriers to effective deployment of this cadre. Deployed principally at urban general hospitals, CHEWS had had little opportunity alongside nurses and midwives to practice the skills they were taught at the School of Health Technology. Many functioned essentially as janitors or security staff at wards, or at best, were used as clerks to register hospital attendees. There was no clarity in the services that should be provided by CHEWs, and their training curriculum had neither been updated nor used as an active tool to shape the skills of students.

These findings led to a concerted effort to develop the CHEW training curriculum at the School of Health Technology to reflect contemporary approaches to community health provision and define more clearly a minimum service package that could be competently delivered by the cadre. The Jigawa state Ministry of Health and the Gunduma Health System Board (the body coordinating management of health services across a cluster of LGAs within the state) developed this minimum service package, mindful of state needs and concerns. The resulting document also was put into quality use and doggedly monitored. Notably, with revisions to the manuals guiding CHEW training, senior CHEWs were accepted as “skilled birth attendants” permitted to attend uncomplicated deliveries.

### Data Sources for Pilot Evaluation

The pilot project was implemented over a 7-month period (February to August 2011). We adopted a quasi-experimental design to evaluate the pilot intervention: routine service data collected through the established Health Management Information Systems (HMIS) were compared for Kadawawa (intervention area) and Kafin Baka (control area), with analysis focusing on the outcomes of health post visits, ANC attendance, and facility-based deliveries. We also compared state HMIS data from the 3-year period of 2008–2010 (before introduction of the pilot) with the 3-year period of 2011–2013 (during and after the pilot) to provide insight into the sustainability of changes following the intense pilot period. The protocol for the study was approved by the Ethics Review Sub-Committee of the Jigawa Operations Research Advisory Committee.

## THE INTERVENTION

Based on findings from the pre-intervention qualitative studies, the planned pilot focused on issues of gender (using female workers), functionality (securing required drugs, equipment and supplies, transportation, etc.), and residence (identifying secure lodgings and infrastructure to support continuity of community engagement). Specific design elements included the following:

- Female CHEWs would be deployed, typically in pairs to support each other, to live and work in remote underserved areas.
- CHEWs would operate from previously dysfunctional health posts in the community (equivalent to the CHPS compounds in northern Ghana).
- CHEWs would be available 24/7 for primary and urgent care visits to the health post.
- They would undertake home visits on rotation with duties at the health post.
- They would be trained to handle normal deliveries; to refer complications of pregnancy and delivery to the hospital in Jahun; and to counsel women on child spacing and provide family planning methods if couples expressed interest, consistent with the cultural sensitivities of the region.
- CHEWs would be trained in the Integrated Management of Childhood Illnesses (IMCI) package and to use the IMCI triage method to refer relevant cases to the nearest PHC.
- They would be provided with the drugs, equipment, and supplies required to provide the basic services specified by the Gunduma Health System Board.
- CHEWs would collaborate with other community-level initiatives such as the Emergency Transport System and community volunteers leading community health dialogues.
- The local community and LGA would assume responsibility for health post renovations, along with provision of necessary furniture and equipment.

Female community health workers were deployed to live and work in remote underserved areas.

### CHEW Selection Criteria

Criteria for selection of CHEWs were developed through discussions with communities. In addition to being female, priority was given to women who were married, ideally already with children born and raised, and who had completed their studies at the Jigawa School of Health Technology, had prior experience working as a CHEW at a health post in the state, were willing to travel to visit isolated hamlets, and had expressed willingness to live and work in a rural remote area without electricity or reliable vehicular access. CHEWs were recruited through advertisements in the local newspaper, at the Jigawa School of Health Technology, and at Jahun General Hospital.

### Training

Selected candidates completed a self-assessment of training needs with respect to the expected duties that were communicated to them. We then provided a 2-week “refresher” training workshop (many of the required skills were formally within their existing scope of practice but had not been operationalized well), using instructors from the Jigawa School of Technology and technical advisors. These CHEWs then joined others from neighboring states that were also piloting community-based service delivery programs for an additional intensive training workshop on key IMCI elements and provision of basic ANC.

Four candidates from Jigawa completed the training and orientation program, 2 of whom were selected for redeployment in Kadawawa in February 2011. The 2 CHEWS rotated between home visits and delivering services at the health post.

### Community-Based Service Delivery Package

The service delivery package at the health post included outpatient, ANC, and immunization services, supervision of normal deliveries, provision of supplementary feeding support, and referral to secondary services. Home visits—structured with respect to an approved protocol and record form—addressed preventive issues, health education, basic primary health care, and identification of family care needs, with referral to the health post or to secondary provision as appropriate (making use of the Emergency Transport System for emergency referrals). The CHEWS also held responsibility for liaising with local health volunteers, such as the nascent cadre of Village Health Workers, to share health education information and identify cases of concern.

### Remuneration

CHEWs received their standard CHEW salary plus a monthly rural residence allowance of 20,000 NGN (about US$100). Salaries for newly trained CHEWs were 38,000–42,000 NGN (US$190–210) per month, while the most senior CHEWs could receive up to 70,000 NGN (about US$350) per month.

The deployed community health workers received a rural residence allowance in additional to their standard salary.

## RESULTS

### Visits to the Health Post

**Figure 1.**
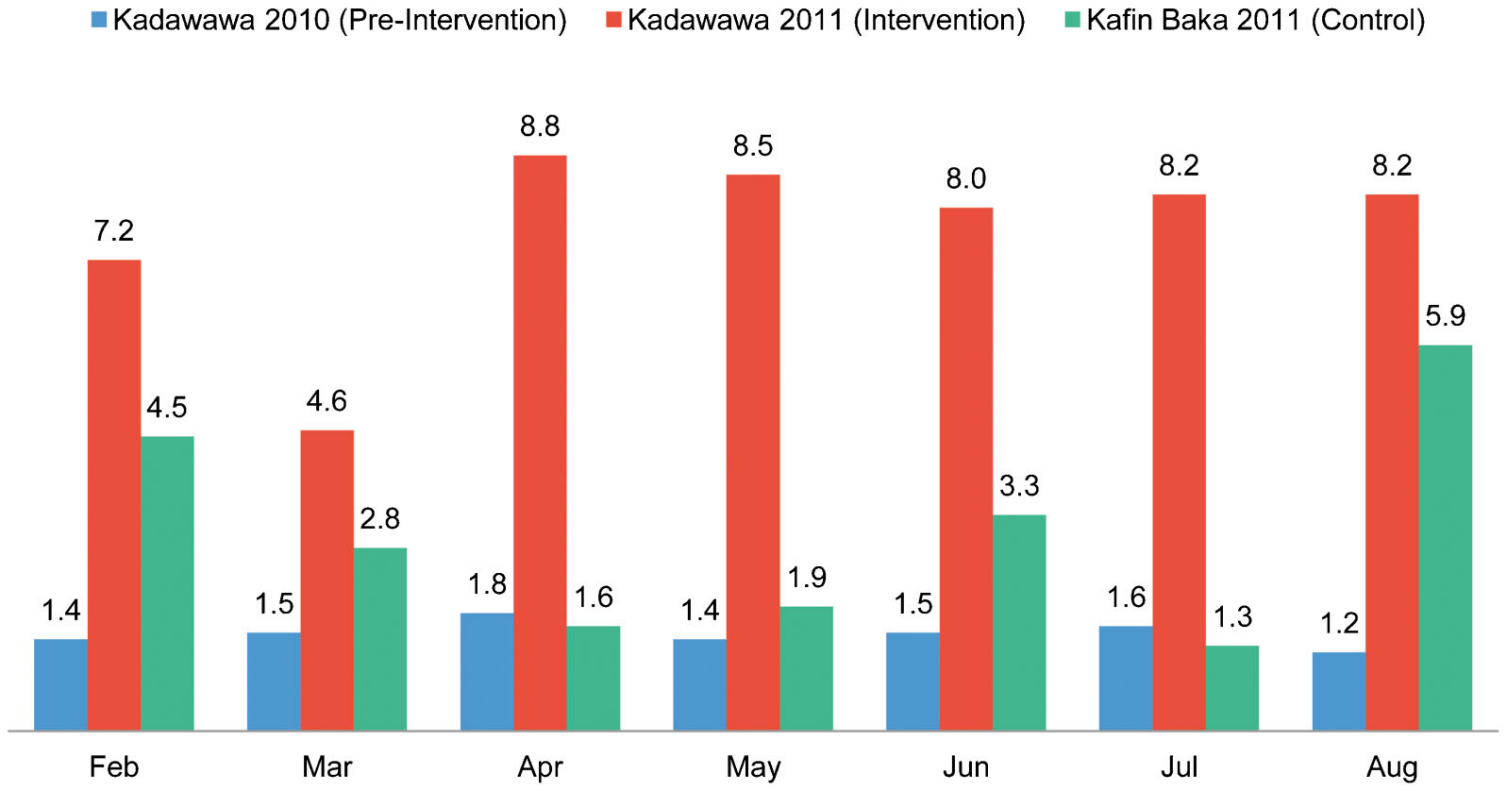
Number of Health Post Visits per 100 Population, Kadawawa Intervention Community (Before and During the Intervention) and Kafin Baka Control Community, Northern Nigeria

In Kadawawa, where the CHEWs were deployed, there were 7.2 health post visits per 100 population in February 2011, the first full month of deployment of the female CHEWs to the health post (Figure 1). In comparison, only 1.4 visits per 100 occurred during February of the preceding year and 4.5 per 100 in the control community clinic at Kafin Baka during the pilot period. After a slight decline in March 2011, health post visit rates in Kadawawa increased to 8.8 per 100 in April 2011 and then remained at or above 8.0 per 100 per month for the rest of the monitoring period. This represented more than a 500% increase of the per capita visit rates from the preceding year, which approximated to the Sphere humanitarian guideline of 1 consultation per capita per year. Although visit rates for Kafin Baka varied substantially from month to month (from 1.3 to 5.9 per 100), visit rates at Kadawawa were between 1.4 and 5.5 times higher than in the control community.

Health post visits per capita increased by more than 500% after deployment of resident female community health workers.

### Antenatal Care

**Figure 2.**
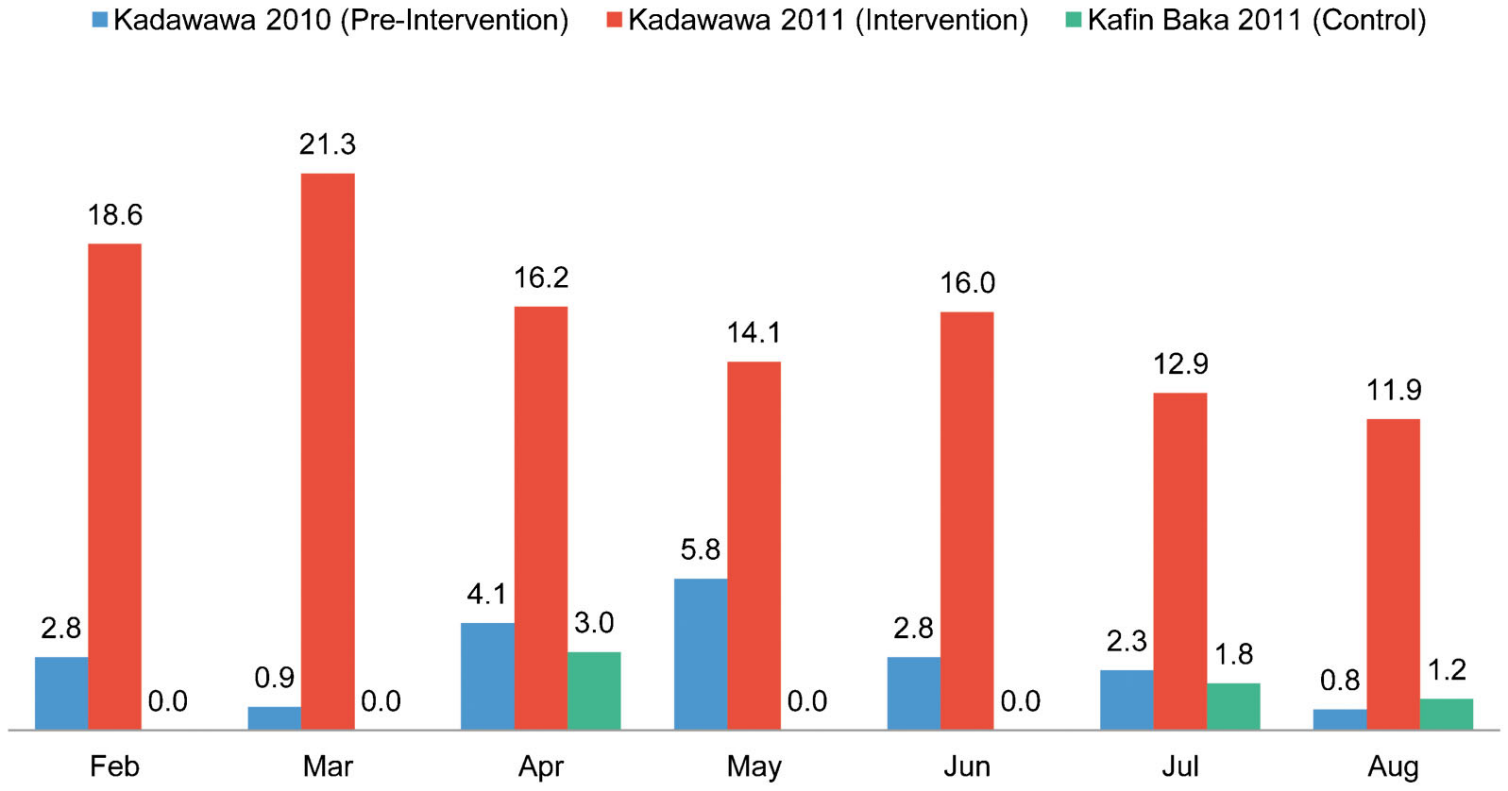
Antenatal Care Attendance Rates per 100 Pregnant Women, Kadawawa Intervention Community (Before and During the Intervention) and Kafin Baka Control Community, Northern Nigeria

Between February and August 2011, the monthly percentage of pregnant women in Kadawawa receiving ANC ranged from 11.9% to 21.3% (Figure 2). In the preceding year, prior to the intervention, coverage had ranged from less than 1% to about 6% in that community. In the control community of Kafin Baka, coverage ranged from only 0% to 3%.

Monthly ANC attendance increased substantially with deployment of resident community health workers.

### Delivery at a Health Facility

**Figure 3.**
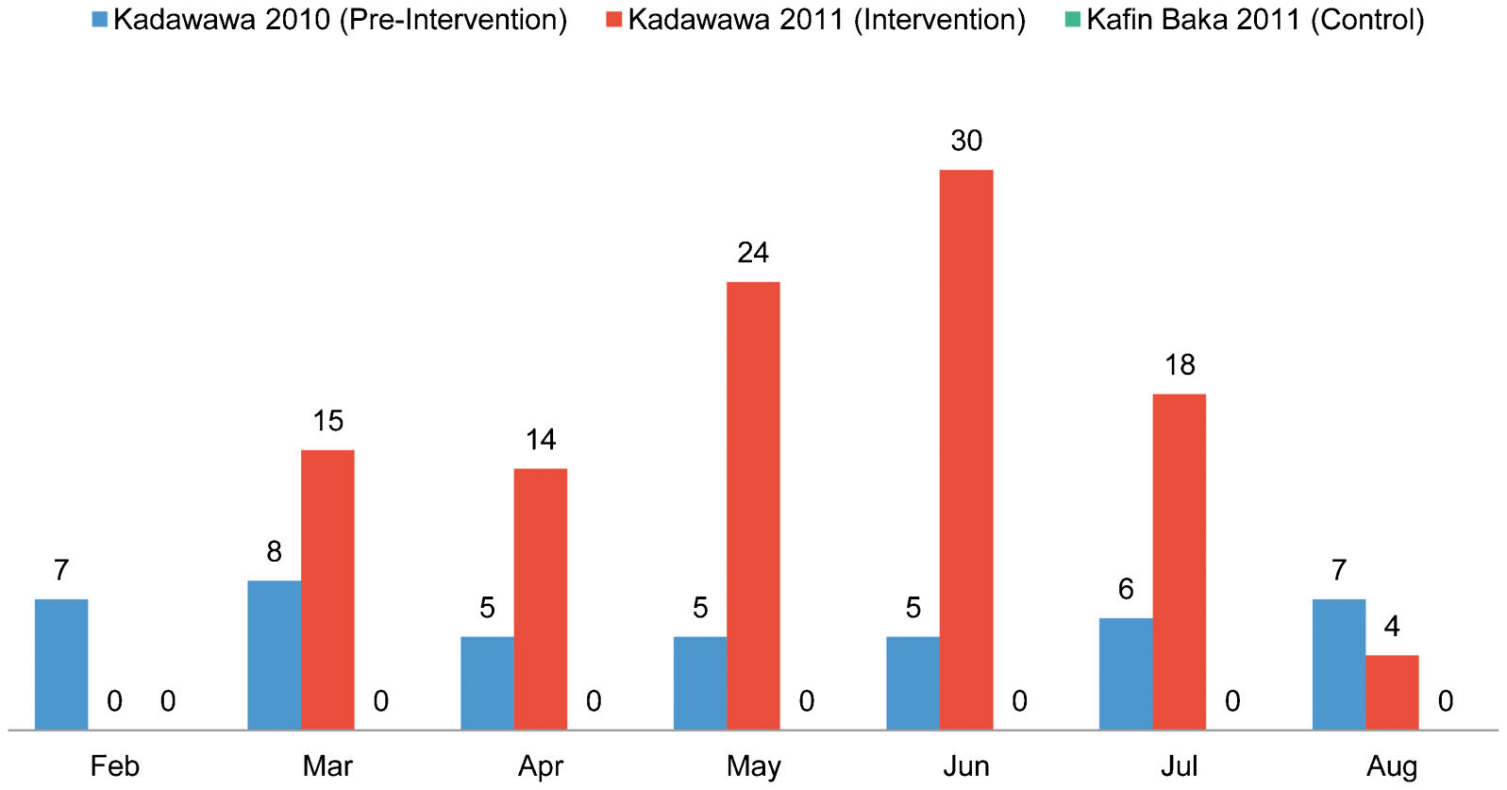
Number of Facility-Based Deliveries by Skilled Birth Attendants, Kadawawa Intervention Community (Before and During the Intervention) and Kafin Baka Control Community, Northern Nigeria

Following deployment of community-based, female CHEWs to Kadawawa, monthly facility-based deliveries by a skilled birth attendant steadily increased to reach 30 deliveries in June 2011 (Figure 3). Although there was some drop-off after this peak, over the 7-month study period facility-based deliveries by a skilled birth attendant more than doubled compared with the preceding year (105 vs. 43, respectively). There were no recorded facility-based deliveries by a skilled birth attendant in the control site of Kafin Baka during the study period.[Fig f04][Fig f05]

Monthly facility-based deliveries increased steadily following deployment of resident female community health workers, reaching 30 deliveries in June 2011.

### Home Visits

By the latter half of 2011, the CHEWs were making on the order of 40 home visits per month to the hamlets in Kadawawa, translating to an annualized home visit rate of over 800 children under 5 years old (approaching 40% of the estimated population of under-5 children).

### Referral

Referrals by traditional birth attendants to CHEWs in Kadawawa for ANC or delivery more than doubled over the study period, according to records provided by CHEWs, suggesting increased confidence and trust in the CHEWs. Referrals from the Kadawawa health post to Jahun General Hospital also increased by over 75% from February to August 2011; prior to the intervention, such referrals had been virtually non-existent.

### Sustainability of Changes

HMIS data indicated that improvements in service utilization in Kadawawa were generally sustained beyond the pilot monitoring period. The following trends were mapped to compare the 3-year period from 2008–2010 before introduction of the service innovation with the 3-year period from 2011–2013 during and after the pilot period:

- Average monthly ANC attendance (first visits): 28–169 vs. 860–1,140, respectively
- Facility-based deliveries: 10–47 deliveries per year vs. 192–260 deliveries per year, respectively
- Immunization: 26–89 children per year fully immunized in the former period vs. a steady increase from 195 in 2001 to 389 in 2012 and 476 in 2013

Service utilization improvements with deployment of resident female community health workers were sustained beyond the pilot period.

Comparable data are not available for the control site of Kafin Baka to consider the influence of wider health systems developments on these figures. However, it seems clear that increases in service utilization in Kadawawa cannot be solely attributed to activities associated with the start-up pilot phase.

Although a detailed cost-benefit analysis was not possible, general indications support the benefits of fielding CHEWs in community-based service delivery to substantially exceed the associated cost of operations. By May 2014, the Kadawawa community had recorded zero maternal and neonatal deaths for 3 consecutive years. PRRINN-MNCH program analysis estimated the overall package of interventions introduced in communities such as Kadawawa to represent an investment of between US$25 and $50 (approximately 5,000–10,000 NGN) per maternal and infant death averted.^22^

## DISCUSSION

After deployment of resident female community health workers to a rural community in northern Nigeria to provide reliable MNCH services at a local health post, 24/7 emergency coverage, and home visits, total health post visits, antenatal care coverage, and facility-based deliveries by a skilled birth attendant rose substantially compared with before the intervention, and were higher than in the control community that did not have resident, female CHEWs. The gains in ANC and skilled birth attendant deliveries were not consistent throughout the pilot intervention period of February–August 2011, but data from 2012 and 2013 indicate a longstanding trend for higher consultation and delivery rates in the intervention community compared with the 3-year period before the intervention.

Key challenges to implementation and scale-up of the community-based service delivery model, however, were identified, including gaps in skills of the CHEWs, training and supervision needs, procurement of supplies, transportation issues for home visits, and recruitment and retention of CHEWs.

### Training and Supervision Needs

It was widely agreed during discussions with a broad range of stakeholder that community-based CHEWs can be an effective cadre to promote safe deliveries and immunization, assess social and support needs, and provide ANC, postnatal care, home visits, sick child case management, growth monitoring and nutritional counseling, and immunization. CHEWs were also considered well placed to provide support for birth spacing and family planning, to recruit and support community volunteers, to liaise with village health committees, and to generally monitor the health situation of a village.

However, during the pilot, we identified gaps in skills of the CHEWs with respect to these roles, and initial or refresher training curricula have subsequently been strengthened to address these gaps, particularly in lifesaving skills, leadership, and communication. Initially, the CHEWs were not trained to perform deliveries, but they reported that in many instances a woman's labor had advanced too far for a safe referral by the time the woman had reached the CHEW's residence or the CHEW encountered the woman during a home visit. Therefore, taking a harm reduction view, the CHEWs were trained to perform deliveries and were provided with delivery kits to be used as needed in these urgent cases.

There was a harm reduction benefit in allowing community health workers to perform deliveries.

Supervision of the deployed CHEWs was challenging during the study period. It had been envisioned that qualified supervisors from urban-based health facilities would be able to balance their work at their own facilities with visits to the staff at Kadawawa. However, personnel shortages and difficulties with travel logistics constrained the frequency and quality of such support.

### Procurement and Transport

During the pilot, the CHEWs experienced irregular drug and non-drug supplies despite commitments made prior to launching the intervention. For example, vaccines were not regularly available partly because of unmet expectations that the Kadawawa health facility would obtain vaccine supplies from another health facility that had a solar refrigerator. However, there were also wider issues, such as periodic national stock-outs of vaccines. A generator for electricity and a refrigerator were eventually provided for vaccine storage at Kadawawa.

The pilot had provided initial “seed stocks” of medicines and supplies, pending the establishment of reliable procurement and supply mechanisms. While regular supply of immunizations and outpatient cards—which are dependent on federal supply sources—remained problematic throughout the study period, CHEWs subsequently received a fairly stable supply of essential drugs, with political, administrative, and financial support from the Gunduma Health System Board and Jahun LGA authorities.

CHEWs experienced difficulties in securing transportation for conducting home visits throughout the study period, especially during the rainy season. These difficulties reflected both logistical and cultural constraints. Local availability of transportation was severely limited, and access to it needed to be negotiated on a daily basis. While male CHEWs potentially could ride motorcycles supplied through the Gunduma Health System Board, there were cultural mores that prevented female CHEWs from having similar access. However, in August 2011, as a result of an advocacy visit to the traditional ruler, the Emir of Dutse, consent was given for female CHEWs to ride “gender-sensitive” motorcycles (which did not expose women's legs while riding) to facilitate their work. Although having no formal legislative role with respect to the health system (which is directed within the state at the level of the Gunduma), the Emir has significant cultural and political influence within this deeply conservative area.

Advocacy with a traditional ruler resulted in allowing the female community health workers to ride motorcycles for their work.

**Figure f05:**
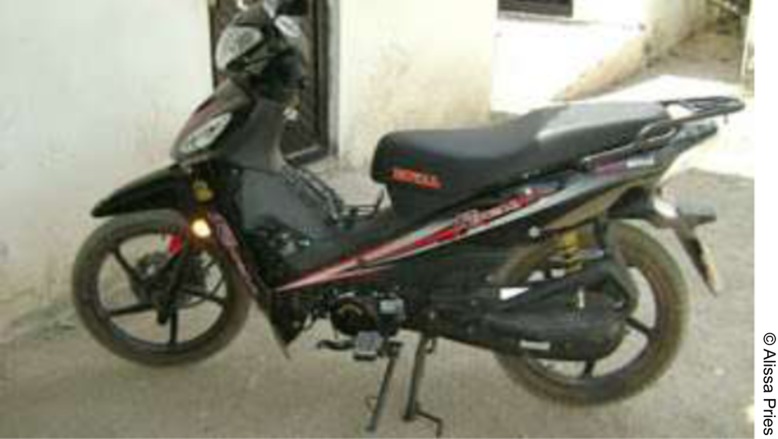
“Gender-sensitive” motorcycles that did not expose women's legs while riding were approved by traditional rulers for use by the female community health workers.

Supply availability and transportation have emerged as recurrent themes in studies of effectiveness and retention of a range of community health worker cadres. For example, Brunie et al., in their study of community health workers in Uganda, found that transportation and commodity stock-outs were the most frequently reported challenges for workers.^14^

### Recruitment and Retention

At the outset of the study, it was clear there were a limited number of female CHEWs in the state who were motivated to live and work in rural areas. The majority of the available CHEWs were married and living in urban areas. Obtaining spousal support to relocate was problematic, and the working environment in rural areas was seen as unattractive. In order to motivate CHEWs to live in rural areas, we offered the deployed CHEWs an additional allowance of 20,000 NGN per month. CHEWs considered the total package (with accommodation, see below) attractive, and by the end of the study period, a number of CHEWs were applying to live and work in the rural areas to which the model was planned for expansion. Further, many Gunduma directors bought into the project by offering unemployed female CHEWs the same 20,000 NGN per month allowance, with accommodation, to live and work in the rural areas. This did not constitute a full employment offer by the councils, but it did help to enlist skilled female staff at the rural areas where 80% of pregnant women in the state reside.

Current practice favors the deployment of unmarried CHEWs for this community-based role, based on the assumption that being single increases the chances of CHEWs agreeing to live and work in a rural area. However, many young, single CHEWs are reported to marry shortly after graduation, and to marry civil servants or other elites who are unlikely to support such deployment. In such circumstances, likely critical incentives (in addition to the rural residence allowance) include employment of female CHEWs as regular health worker staff (rather than as contract staff) and limiting the minimum period of service, for example, to 2 years. Permission for spousal and other family member visits was an important element of support for the female CHEWs in Kadawawa, enabling them to stay in their posts longer and reducing their feelings of isolation from their families. Similarly, Abbey et al. report approval of the community and the health worker's immediate family as the most significant factors in predicting retention of community-based workers in Dangme West district in Ghana.^23^ Over the long term, improving the quality of and gender parity in education and training is required to increase the pool of women from remote rural areas who are equipped to serve as health workers in their home areas (a major focus of the DFID-funded Women for Women Health initiative).^24^

A precondition for deploying and retaining female CHEWs was provision of adequate housing accommodations. To support retention of CHEWs in Kadawawa beyond the study period, part of the existing health facility was rehabilitated to create self-contained units (a bedroom and a sitting room) to accommodate the resident CHEWs. A kitchen was also created and toilets were rehabilitated, with provision of an external water tank that also supplied water to the CHEW consulting rooms. During the study period, the CHEWs in Kadawawa had been accommodated in the home of a local leader who was supportive of the intervention. Such community engagement in the model was vital in a context where delays and instability threaten service developments when dependent on formal governmental channels alone.

Ensuring adequate housing accommodations and providing an extra rural residence allowance were critical in recruiting and retaining rural-based community health workers.

Finally, we found that deploying the CHEWs in pairs helped avoid feelings of isolation, which can help with retention and recruitment of health workers. In addition, they provided support to each other in their work. For example, the CHEWs rotated duties for home visits and staffing the health post.

### Scale-Up of the Model

Jigawa state health officials presented the successful findings from the Kadawawa pilot study to the State Assembly and advocated employment of more female CHEWs to scale-up the service model. Their efforts were successful, and the Gunduma Health System Board subsequently budgeted for recruitment of additional female CHEWs. Communities and associated facilities were identified for the preliminary phase of expansion in 2013 to up to 30 locations, with associated plans for establishing CHEW residential accommodations. This entailed an allocation of an additional 20 million NGN in the Gunduma Health System Board budget, signaling endorsement of the pilot model as an effective and scalable strategy across the state.

Fifteen new health posts were refurbished and equipped, and an additional 60 female CHEWs were hired to expand the community-based service delivery model into 30 new sites. In addition, 30 “gender-sensitive” motorcycles were procured for female CHEWs to facilitate their home visits. Some of the budgeted funds were also used to purchase material incentives (baby wipes, soap, birth towels for newborn babies, wrappers, delivery kits) for pregnant women who delivered at the refurbished health facilities.

State health officials expanded the community-based service delivery model to an additional 30 sites.

To ensure sustainability, the State Ministry of Health, the Ministry of Women Affairs and Social Development, the Gunduma Councils, and the Ministry of Local Government established a pooled fund to support community-based service delivery activities. In reflecting on the factors that enabled formulation and implementation of the pilot community-based service delivery model, and supported its subsequent planned scale-up, the value of an enabling governance environment should not be ignored. Since 2007, management of health facilities in Jigawa has been delegated from the State Ministry of Health to the Gunduma Health System Board, which then devolves services to the 9 Gunduma Councils.^25^ The director of a Gunduma Council is thus the administrative head of both PHCs and general hospitals under that council, allowing an unusual degree of coordinated planning. Between 2009 and 2012, Jigawa nearly doubled its state health expenditure,^22^ with 90% or more of health budget allocations fully released each year.

## CONCLUSION

A pilot deploying female community health workers to residential postings in a rural area of northern Nigeria increased health post visits, antenatal care coverage, and facility-based deliveries by a skilled birth attendant. The positive findings mobilized authorities to scale-up this model within the state. Key lessons learned include the harm reduction benefit of allowing community health workers to perform deliveries, the need to ensure regular supplies and sufficient personnel to provide supportive supervision, the importance of arranging culturally sensitive transportation means to conduct home visits, and the critical need to provide health workers an additional rural residence allowance to recruit and retain them.

## Notes

**Peer Reviewed**

## References

[1] Hogan MC Foreman KJ Naghavi M Ahn SY Wang M Makela SM. Maternal mortality for 181 countries, 1980–2008: a systematic analysis of progress towards Millennium Development Goal 5. Lancet. 2010;375(9726): 1609–1623. 10.1016/S0140-6736(10)60518-1. 20382417

[2] National Population Commission (NPC) [Nigeria]; ICF International. Nigeria demographic and health survey 2013. Abuja (Nigeria): NPC; 2014 Co-published by ICF International. Available from: http://dhsprogram.com/pubs/pdf/FR293/FR293.pdf

[3] United Nations Children's Fund (UNICEF). Countdown to 2015: tracking progress in maternal, newborn and child survival: 2008 report. New York: UNICEF; 2008 Available from: http://www.countdown2015mnch.org/reports-and-articles/previous-reports/2008

[4] Center for Reproductive Rights; Women Advocates Research and Documentation Centre. Broken promises: human rights, accountability, and maternal death in Nigeria. New York: Centre for Reproductive Rights; 2008 Available from: http://reproductiverights.org/sites/crr.civicactions.net/files/documents/pub_nigeria2.pdf

[5] Federal Ministry of Health [Nigeria]. Saving newborn lives in Nigeria: newborn health in the context of the Integrated Maternal Newborn and Child Health Strategy. Abuja (Nigeria): The Ministry; 2009 Co-published by Save the Children.

[6] Partnership for Revitalizing Routine Immunization in Northern Nigeria (PRRINN). Baseline survey. Kano (Nigeria): PRRINN; 2007.

[7] Gilmore B McAuliffe E. Effectiveness of community health workers delivering preventive interventions for maternal and child health in low- and middle-income countries: a systematic review. BMC Public Health. 2013;13(1): 847. 10.1186/1471-2458-13-847. 24034792 PMC3848754

[8] Koon AD Goudge J Norris SA. A review of generalist and specialist community health workers for delivering adolescent health services in sub-Saharan Africa. Hum Resour Health. 2013;11:54. 10.1186/1478-4491-11-54. 24160988 PMC3874771

[9] Bamisaiye A Olukoya A Ekunwe EO Abosede OA. A village health worker programme in Nigeria. World Health Forum. 1989;10(3–4): 386–392. 2637712

[10] Fagbule D Kalu A. Case management by community health workers of children with acute respiratory infections: implications for national ARI control programme. J Trop Med Hyg. 1995;98(4): 241–246. 7636920

[11] Hermann K Van Damme W Pariyo GW Schouten E Assefa Y Cirera A. Community health workers for ART in sub-Saharan Africa: learning from experience–capitalizing on new opportunities. Hum Resour Health. 2009;7:31. 10.1186/1478-4491-7-31. 19358701 PMC2672918

[12] Kironde S Bajunirwe F. Lay workers in directly observed treatment (DOT) programmes for tuberculosis in high burden settings: should they be paid? A review of behavioural perspectives. Afr Health Sci. 2002;2(2): 73–78. 12789106 PMC2141572

[13] Onwujekwe O Uzochukwu B Ojukwu J Dike N Shu E. Feasibility of a community health worker strategy for providing near and appropriate treatment of malaria in southeast Nigeria: an analysis of activities, costs and outcomes. Acta Trop. 2007;101(2): 95–105. 10.1016/j.actatropica.2006.07.013. 17270139

[14] Brunie A Wamala-Mucheri P Otterness C. Keeping community health workers in Uganda motivated: key challenges, facilitators, and preferred program inputs. Glob Health Sci Pract. 2014;2(1): 103–116. 10.9745/GHSP-D-13-00140. 25276566 PMC4168609

[15] Mumtaz Z Salway S Waseem M Umer N. Gender-based barriers to primary health care provision in Pakistan: the experience of female providers. Health Policy Plan. 2003;18(3): 261–269. 10.1093/heapol/czg032. 12917267

[16] Bhutta ZA Lassi ZS Pariyo G Huicho L Global experience of community health workers for delivery of health related Millennium Development Goals: a systematic review, country case studies, and recommendations for integration into national health systems. Geneva: World Health Organization, Global Health Workforce Alliance; 2010 Available from: http://www.who.int/workforcealliance/knowledge/publications/alliance/Global_CHW_web.pdf

[17] Jaskiewicz W Tulenko K. Increasing community health worker productivity and effectiveness: a review of the influence of the work environment. Hum Resour Health. 2012;10:38. 10.1186/1478-4491-10-38. 23017131 PMC3472248

[18] Doctor HV Findley SE Ager A Cometto G Afenyadu GY Adamu F. Using community-based research to shape the design and delivery of maternal health services in Northern Nigeria. Reprod Health Matters. 2012;20(39): 104–112. 10.1016/S0968-8080(12)39615-8. 22789087

[19] Awoonor-Williams JK Feinglass ES Tobey R Vaughan-Smith MN Nyonator FK Jones TC. Bridging the gap between evidence-based innovation and national health-sector reform in Ghana. Stud Fam Plann. 2004;35(3): 161–177. 10.1111/j.1728-4465.2004.00020.x. 15511060

[20] National Population Commission (NPC) [Nigeria]. State population projections. Abuja (Nigeria): NPC; 2006.

[21] Uzondu C Petit L Pries A Report on pre-intervention qualitative studies at Kadawawa and Takalafiya. Presented to the Jigawa State Operations Research Advisory Committee. Dutse, Jigawa State (Nigeria); PRRINN-MNCH Programme ; 2010.

[22] Partnership for Reviving Routine Immunisation in Northern Nigeria-Maternal Newborn and Child Health Initiative (PRRINN-MNCH). Better maternal, newborn & child health in Northern Nigeria: final report 2013. East Sussex (UK): PRRINN-MNCH; 2013 Available from: http://www.prrinn-mnch.org/documents/PRRINN-MNCHFinalReport2013.pdf

[23] Abbey M Bartholomew LK Nonvignon J Chinbuah MA Pappoe M Gyapong M. Factors related to retention of community health workers in a trial on community-based management of fever in children under 5 years in the Dangme West District of Ghana. Int Health. 2014;6(2): 99–105. 10.1093/inthealth/ihu007. 24532651

[24] Department for International Development of the United Kingdom (DFID). Women for Women Health Initiative in Nigeria: contract award notice. East Kilbride (UK): DFID; 2012 Available from: https://www.devex.com/en/contracts/women-for-women-health-initiative-in-nigeria

[25] Ali-Akpajiak S The Gunduma story: emerging health system architecture to reform a disintegrated system in Jigawa State of Nigeria. Kano (Nigeria): PRRINN-MNCH; 2010 Available from: http://www.prrinn-mnch.org/documents/ExecutiveSummary_Gundumastory_final_Jun10.pdf

